# From Thrombectomy to Dialysis: Case Report of Acute Kidney Injury Due to AngioJet® in a Patient With Deep Venous Thrombosis

**DOI:** 10.7759/cureus.91109

**Published:** 2025-08-27

**Authors:** Michel S Castaño Cardozo, Claudia Valentina Valderrama Teran, Alejandro Valderrama Teran, Luis Alfonso Valderrama Cometa

**Affiliations:** 1 Department of Nephrology, Centro Médico Imbanaco, Cali, COL; 2 Department of Medicine, Instituto Colombiano de Estudios Superiores de Incolda (ICESI) University, Cali, COL; 3 Department of Medicine, Pontificia Universidad Javeriana Seccional Cali, Cali, COL

**Keywords:** acute kidney injury, angiojet, deep vein thrombosis (dvt), hemoglobinuria, mechanical thrombectomy

## Abstract

Deep vein thrombosis (DVT) is a common venous thrombotic disorder. Current treatment strategies focus on anticoagulation and pharmacomechanical thrombectomy technologies; however, associated complications must be considered. Acute kidney injury (AKI) secondary to hemoglobinuria requiring renal replacement therapy is a rare yet serious complication of mechanical thrombectomy with the AngioJet® rheolytic thrombectomy system. This report describes the case of a 47-year-old male patient who developed AKI, hemoglobinuria, and metabolic acidosis within hours of the procedure. Despite fluid administration and urine alkalinization, renal replacement therapy was required; renal function subsequently recovered. This case illustrates the risk of AKI from hemoglobinuria associated with mechanical thrombectomy, plausibly mediated by hemolysis and oxidative stress leading to renal tubular obstruction. Close renal monitoring and individualized risk‑factor assessment facilitate early recognition and timely intervention, potentially mitigating the risk of chronic kidney disease.

## Introduction

Deep vein thrombosis (DVT) is a common venous thromboembolic disorder primarily affecting the lower limb, typically originating in deep calf veins and propagating proximally [1]. DVT affects 10 million people annually, ranking as the third most common vascular disease. The incidence of DVT increases with age and is linked to factors such as obesity, cancer, and surgical procedures [2]. The primary treatment for DVT involves anticoagulants to prevent clot growth and reduce complications like pulmonary embolism (PE), symptomatic nonfatal PE, and recurrent venous thromboembolism (VTE) [3]. Catheter-directed thrombolysis (CDT) is used selectively in acute iliofemoral deep vein thrombosis cases and may provide short‑term symptom relief, improved quality of life, and enhanced thrombus removal [2,3].

The AngioJet® Rheolytic Thrombectomy Catheter System (Boston Scientific, Marlborough, MA, USA) combines catheter‑based and mechanical technology; its “Power Pulse” mode delivers high‑pressure saline jets that fragment thrombus and facilitate aspiration [4-6]. The use of this system has been associated with a reduced risk of post‑thrombotic syndrome in selected populations [7]. The use of pharmaco-mechanical thrombectomy (PMT) has been associated with various complications. Renal involvement, including the need for renal replacement therapy, has been reported as one such complication [4,8]. Additionally, hemoglobinuria has been reported post‑procedure and attributed to device‑related hemolysis during AngioJet® rheolytic thrombectomy [6]. 

This report presents a case of acute kidney injury (AKI) secondary to hemoglobinuria requiring renal replacement therapy after mechanical thrombectomy with the AngioJet® Thrombectomy Catheter System, underscoring this rare yet potentially serious complication and its implications for renal function.

## Case presentation

A 47‑year‑old male patient presented to the emergency department with edema, warmth, erythema, and pain in the right lower extremity, worsened by ambulation and without fever or recent trauma. His history was notable for knee arthroscopy performed 64 days earlier, which included partial medial meniscectomy (25%) and medial meniscal repair for a two-layer tear, without subsequent administration of thromboprophylaxis.

**Figure 1:**
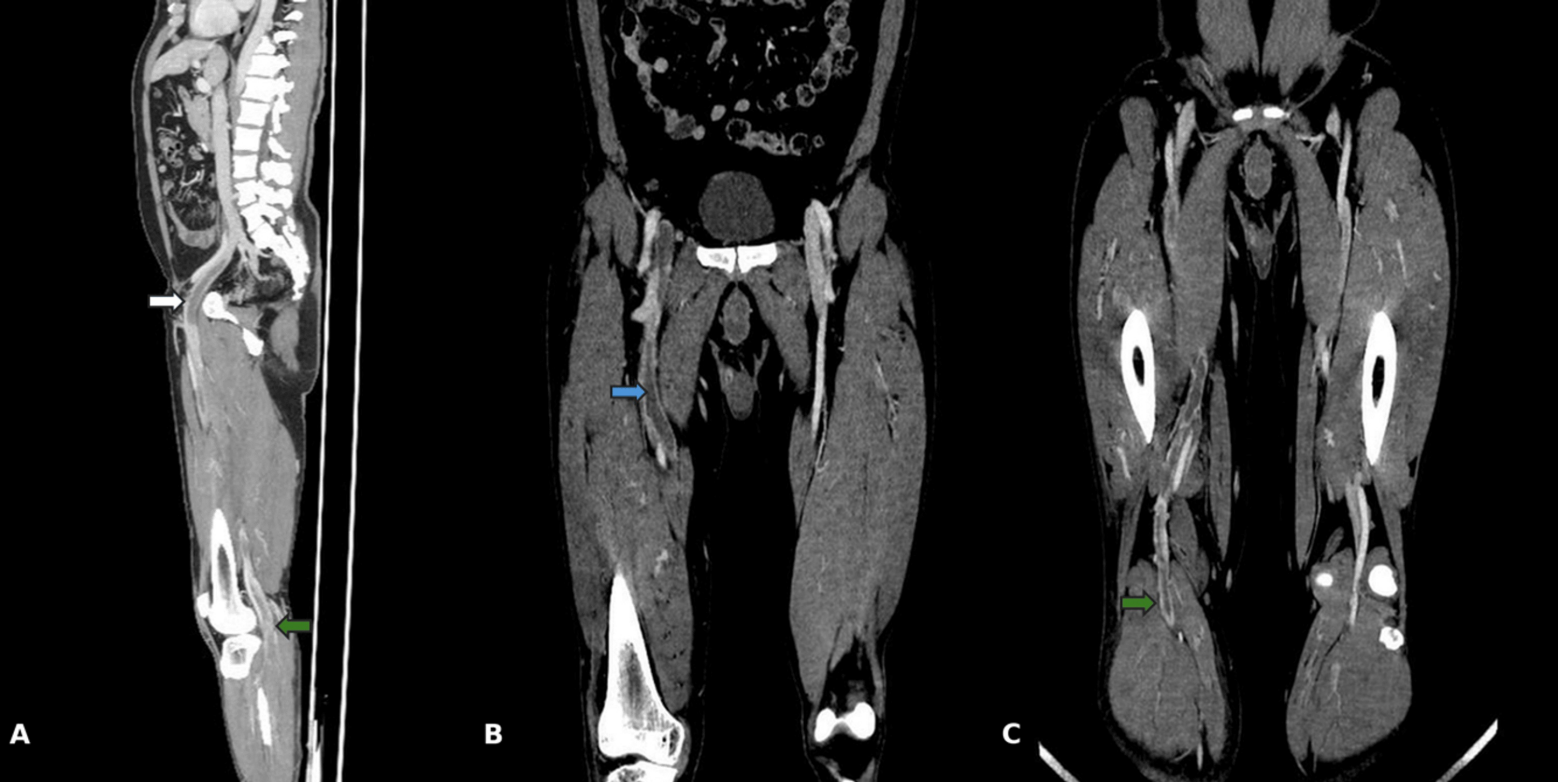
Contrast‑enhanced CT angiography demonstrating extensive right‑sided iliofemoral DVT with distal extension (A) Sagittal reconstruction shows a central low‑attenuation intraluminal filling defect in the right external iliac vein (white arrow) with distal propagation affecting the popliteal segment (green arrow). (B) Coronal reconstruction at the groin demonstrates an intraluminal filling defect in the right external iliac/common femoral vein segment (blue arrow). (C) Coronal reconstruction at the thigh/knee level shows continuation of the filling defect into the femoral–popliteal segment (green arrow), consistent with extensive deep vein thrombosis of the right lower extremity. Abbreviations: CT, computed tomography; DVT, deep vein thrombosis.

On physical examination on admission, vital signs were within normal limits, with evident edema and erythema from the knee to the ankle of the right lower extremity. Initial laboratory results showed a white blood cell count of 7,930/µL (reference 4,230-9,070/µL), hemoglobin at 14.5 g/dL (reference 13.7-17.5 g/dL), and serum creatinine at 0.95 mg/dL (reference 0.67-1.17 mg/dL). Doppler venous ultrasonography demonstrated extensive thrombosis, prompting contrast‑enhanced CT image. CT angiography demonstrated an extensive central low‑attenuation intraluminal filling defect within the right external iliac vein with extension to the common femoral, deep femoral, and superficial femoral veins, with continuation into the popliteal vein, consistent with extensive DVT of the right lower extremity (Figure 1).

The patient underwent mechanical thrombectomy of the right iliofemoral segment with AngioJet®, and post‑procedural imaging showed complete removal of thrombus in this segment. A 0.9% saline solution was administered at 50 mL/h. However, a few hours post-procedure, the urine became dark. The patient developed metabolic acidosis, with urinalysis revealing hemoglobin at 250/µL (normally negative) in the absence of red blood cells, and a serum creatine phosphokinase (CPK) of 3,161 U/L (reference 20-200 U/L). Despite preserved urine output, after 48 hours there was an elevation in serum creatinine and blood urea nitrogen (BUN) to values consistent with Acute Kidney Injury Network (AKIN) stage III, accompanied by a 2 g/dL decline in hemoglobin and a lactate dehydrogenase (LDH) of 536 U/L (reference 135-225 U/L). The intravenous fluid rate was increased to 100 mL/h. Renal ultrasonography showed findings compatible with non‑obstructive acute nephropathy and elevated intrarenal resistive indices in all evaluated segments of both renal arteries. Urine alkalinization was pursued and achieved with bicarbonate‑containing intravenous fluids, and furosemide was administered for diuresis.

**Figure 2:**
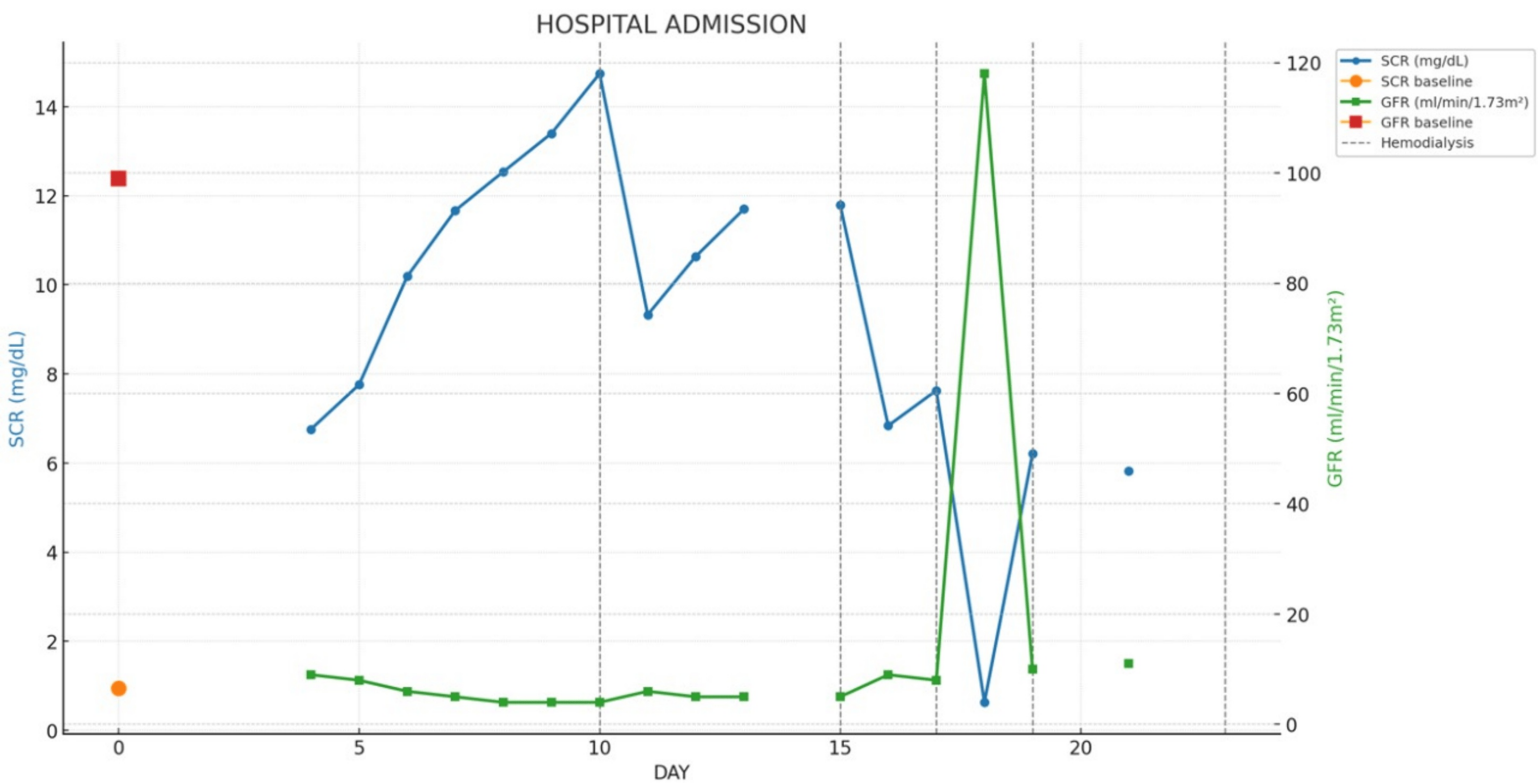
In‑Hospital Renal Function Trajectory Time series of SCR (blue; left y‑axis) and eGFR (green; right y‑axis) from baseline through hospitalization. Orange circle and red square denote baseline SCR and baseline eGFR, respectively. The vertical dashed lines indicate hemodialysis sessions. Creatinine increased after the procedure with a nadir in eGFR, followed by improvement after initiation of intermittent hemodialysis. Abbreviations: eGFR, estimated glomerular filtration rate; SCR, serum creatinine.

Despite these interventions, the patient developed oliguria eight days post‑procedure, with signs of volume overload, positive fluid balance, and persistent rises in serum creatinine to 14.7 mg/dL and BUN to 88.6 mg/dL (reference 6-20 mg/dL). Hemodialysis was initiated, with sessions performed every two days (Figure 2).

**Figure 3:**
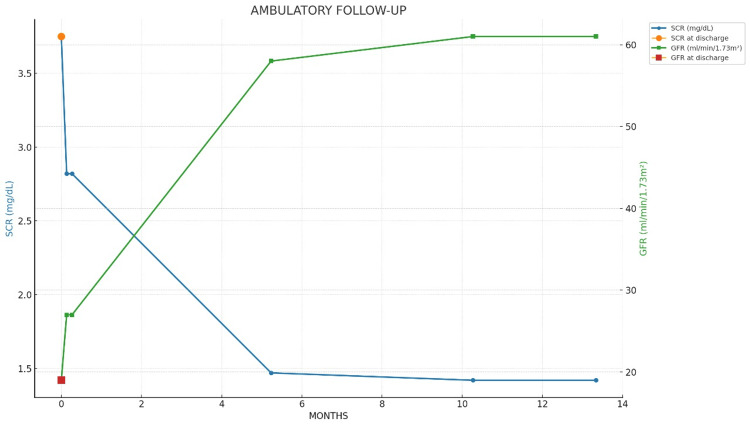
Outpatient Renal Function Recovery Time‑series plot showing SCr (blue; left y‑axis) and eGFR (green; right y‑axis) during outpatient follow‑up from discharge to 12 months. Orange circle and red square indicate discharge values for SCR and eGFR, respectively. Values show progressive decline in SCr and rise in eGFR with stabilization by month 6. Abbreviations: eGFR, estimated glomerular filtration rate; SCr, serum creatinine.

At one month post‑procedure, the estimated glomerular filtration rate was 44 mL/min/1.73 m², consistent with renal recovery and no further dialysis requirement (Figure 3).

## Discussion

This report describes a patient with right lower‑extremity DVT who underwent mechanical thrombectomy with the AngioJet® Rheolytic Thrombectomy Catheter System. Despite apparent thrombus clearance, the patient developed hemoglobinuria and AKI, ultimately requiring hemodialysis. The case illustrates potential complications of pharmacomechanical thrombectomy, particularly AKI following rheolytic thrombectomy.

Cases of AKI have been reported after use of the AngioJet® rheolytic thrombectomy system. The PEARL (Peripheral Use of AngioJet Rheolytic Thrombectomy with a Variety of Catheter Lengths) registry reported that three patients developed AKI requiring dialysis after the procedure [9]. However, this study did not describe possible etiologies or preventive strategies for this complication.

A case similar to ours was reported by Mathews et al. They described a patient with extensive lower‑extremity DVT who underwent PMT and subsequently developed severe hemoglobinuria‑related AKI requiring dialysis for five months; the authors suggested a possible association with a high thrombus burden [10]. These observations support further investigation into risk factors for AKI in this setting.

Shen et al. [11] investigated the risk of postoperative AKI in patients with acute iliofemoral DVT who underwent PMT using AngioJet® or CDT. AngioJet®‑assisted PMT was associated with higher odds of AKI compared with CDT (OR 2.8; p=0.022), with an incidence of 22.8% in the AngioJet® group within a cohort of 198 patients. Additionally, a history of major surgery within the preceding three months emerged as an independent risk factor for postoperative AKI [11], a factor present in the current case that may have contributed to the outcome.

PMT is often associated with hemoglobinuria, which may compromise kidney function and precipitate AKI. These devices induce intravascular hemolysis through mechanical rupture of erythrocytes during the intervention, increasing plasma‑free hemoglobin. Hemoglobin is a tetrameric protein composed by a porphyrin ring containing heme prosthetic groups (bound to ferrous iron). Once filtered, hemoglobin/heme is reabsorbed via endocytosis by megalin/cubilin receptor complex in proximal tubular cells. This process promotes reactive oxygen species (ROS) generation and direct tubular cytotoxicity, triggering oxidative stress, mitochondrial injury, and cell death. Inflammatory cytokine release has also been described, further contributing to tubular injury, as documented in renal biopsies of affected patients [8,12]. Although plasma-free hemoglobin levels were not measured in this case, the concomitant elevation of LDH and reduction in hemoglobin concentration were indicative of hemolysis.

Conversely, filtered hemoglobin heterodimers may precipitate and form aggregates with Tamm-Horsfall protein (uromodulin) within the tubular lumen, generating hemoglobin‑containing casts (hematic cast) that obstruct distal nephron segments and contribute to tubular injury [13]. Experimental studies also indicate that podocytes internalize hemoglobin, with subsequent increases in reactive oxygen species (ROS), premature apoptosis, and glomerular proteinuria, thereby contributing to nephrotoxicity [14].

It should be noted that there was a cumulative effect of various risk factors for the development of AKI including rhabdomyolysis and contrast exposure, which together with hemoglobinuria, exert a significant impact on renal function.

Although AngioJet® use has been identified as a risk factor for hemoglobinuria, some studies suggest that nephroprotective strategies, operator expertise, and periprocedural optimization may mitigate the risk of AKI. The reported measures include limiting thrombus aspiration time, maintaining adequate and timely hydration, urine alkalinization, and a comprehensive pre‑procedural evaluation [15].

## Conclusions

This case illustrates that hemoglobinuria and AKI are potentially serious complications following AngioJet®‑assisted pharmacomechanical thrombectomy, particularly in the setting of extensive thrombus burden and prolonged procedure times, which increase exposure to contrast media and predispose to the development of rhabdomyolysis. These observations support close monitoring of renal function and the timely initiation of renal replacement therapy in patients who develop post‑procedural renal impairment. Additionally, these observations support careful appraisal of individual risk factors and the risk-benefit balance when AngioJet®‑assisted pharmacomechanical thrombectomy is contemplated.
